# Precision-cut tumor slices: a biomimetic tool for accelerating oncological development from basic research to clinical translation

**DOI:** 10.3389/fphys.2026.1848297

**Published:** 2026-09-21

**Authors:** Xiaojun Lei, Song Bao, Dong Jin, Jinwen Zhou, Tao Guo, Jing Kong

**Affiliations:** 1 School and Hospital of Stomatology, Dalian Medical University, Dalian, Liaoning, China; 2 The First Affiliated Hospital, Dalian Medical University, Dalian, Liaoning, China

**Keywords:** drug screening, precision medicine, precision-cut tumor slices, preclinical model, tumor microenvironment

## Abstract

Faithfully recapitulating the complex pathology of human tumors is a critical requirement for effective cancer research. However, animal models are limited by interspecies differences, and traditional *in vitro* systems often fail to maintain the native tissue architecture. Precision-cut tissue slices that retain the original tissue’s histology, cellular heterogeneity, and physiological functions offer a compelling alternative. Cultured precision-cut tumor slices (PCTS) leverage these advantages to provide a highly representative model for investigating tumor biology and advancing therapeutic development. This review details PCTS, from tissue preparation and optimized culture conditions to their diverse applications in mechanistic research and preclinical drug evaluations.

## 1 Introduction

In recent decades, invasive and noninvasive techniques for monitoring malignant tumors have advanced substantially. Molecular diagnostics, immunotherapy, and small-molecule inhibitors are being increasingly used in clinical practice. Despite these developments, cancer morbidity and mortality rates remain unacceptably high (Wagle et al., 2025; Wu et al., 2026c). These challenges stem largely from tumor heterogeneity, which is driven by dynamic interactions within the tumor microenvironment (TME). The TME comprises carcinoma-associated fibroblasts, immune cells, cancer stem cells, endothelial cells, tumor-derived factors, and the extracellular matrix (ECM) (Chen et al., 2024). Critically, key tumor behaviors, including initiation, invasion, metastasis, and therapeutic resistance, depend on the TME (Wu and Dai, 2017; Ribeiro Franco et al., 2020). Experimental systems are being fundamentally redesigned to better model the TME for drug screening and preclinical studies.

Cell culture systems are fundamental tools for cancer research but fail to fully replicate the complex spatial architecture and organization observed *in vivo* (Abuwatfa et al., 2024). Moreover, continuous monitoring of the entire pathological progression from healthy tissue to precancerous lesions to invasive malignancy is essential for understanding tumorigenesis. This process is inherently unpredictable in humans, making sequential observations challenging (Habanjar et al., 2021). Tumor organoids have been developed to address this issue. Organoids are typically derived clonally from the primary human tumor tissue, pluripotent stem cells, or adult stem cells and can recapitulate key histopathological features and heterogeneity of the original tumor (Tuveson and Clevers, 2019; Wu et al., 2026b). A significant limitation of tumor organoids, however, is their extended generation time and nonstandardized processing (LeSavage et al., 2022). Animal models, including transplant models (Guerin et al., 2020), carcinogen-induced models (Sewduth and Georgelou, 2024) and genetically engineered mouse models (GEMMs) (Kersten et al., 2017), remain indispensable for preclinical applications. However, concerns persist regarding their ability to faithfully recapitulate the pathogenic processes specific to human cancers.

Precision-cut tissue slices (PTS) are thin, uniform explants derived from human or animal organs. They retain more complex histological structures and physiological functions *in vitro* (Siwczak et al., 2023). The application of tissue slices in biochemistry dates back to the 1920s, notably in early metabolic studies (Warburg et al., 1927). Research utilizing this technique advanced significantly in the 1980s following Krumdieck et al.’s invention of a specialized slicer capable of reproducibly generating tissue slices with a precise thickness (Krumdieck et al., 1980). The development of suitable tissue embedding materials represents another essential advancement in PTS methodology. In 1987, Fisher and colleagues pioneered the agarose-filled embedding method, successfully maintaining 2 mm-thick cross-sections of lung lobes that were viable for 4 to 6 weeks (Placke and Fisher, 1987). The successful generation and culture of PTS have been reported for numerous organs, including the brain, heart, intestine, kidney, liver, lung, prostate, and spleen (Parrish et al., 2002; de Graaf et al., 2010; Brandenburger et al., 2012; Haque et al., 2013; Mattei et al., 2015; Bigaeva et al., 2020; Koziol-White et al., 2024).

Compared with other available tumor models, precision-cut tumor slices (PCTS) better preserve the integrity of the TME, maintain genetic diversity, and more faithfully recapitulate drug responses and resistance (**Table 1**). Consequently, PCTS have garnered increasing attention in oncology research. More importantly, their eventual translation into clinical diagnosis and treatment parallels the developmental path of other model systems and remains a priority. This review covers key methodological aspects of PCTS preparation and culture conditions, with an emphasis on recent applications in basic cancer biology, therapeutic development, and diagnostics. We also discuss current technical and logistical challenges in PCTS handling and application, from which we derive perspectives aimed at advancing their future clinical translation.

**Table 1:** The difference between PCTS and other three-dimensional cell culture systems.

Models	2D cell culture	Tumor spheroids	Tumor organoids	Patient-derived xenografts (PDX)	Precision cut tumor slices (PCTS)	Organ-on-a-chip
Tissue Source	Immortalized tumor cell lines	Immortalized tumor cell lines or purified primary tumor cells	Immortalized cell lines or patient tumor tissues	Fresh patient tumor tissue resected during surgery	Fresh tumorous tissues from patients or animals	Artificial co-seeding of tumor, stromal and immune cells
Establishment Time	1–2 days	1–3 days	2–4 weeks	1–6 months	< 1 day	1–2 weeks
Culture Cost	Low	Low	Moderate	High	Low	High
Operation Difficulty	Low	Low	High	Moderate	Moderate	High
TME Completeness	Absent; no stromal and immune cells	Partial; no complete stromal and circulating immune cells	Partial; no functional immune cells and vascular structure	Complete functional ecosystem; with interspecies differences	Intact human stroma; no circulating immune cells	Partial; no native tissue spatial structure
Reproducibility	High	High	Moderate	Moderate	Moderate	Moderate
Genetic Heterogeneity	Low	Low	Moderate	Low	High	Moderate
Drug Sensitivity*	Low	Moderate	Moderate	High	High	Based on cultivation methods
Drug Resistance	Low	Moderate	Moderate	High	High	Based on cultivation methods
Core Applications	Basic research Cytotoxicity assay	Basic research Drug screening Precision medicine Drug delivery	Basic research Drug screening Chemoradiotherapy Immunotherapy	Basic research Personalized therapy Immunotherapy Drug screening	Basic research Drug screening Non-pharmacological therapy	Basic research Drug screening Drug toxicity Personalized therapy

*Drug sensitivity is evaluated by the consistency between model responses and *in vivo* human clinical outcomes. Comparative summary of core properties and applicable scenarios of precision cut tumor slices (PCTS), 2D Cell Culture, tumor spheroids, tumor organoids, patient-derived xenografts (PDX) and tumor organ-on-a-chip systems. Multiple key indicators are systematically evaluated, including tissue source, establishment time, culture cost, operation difficulty, TME completeness, reproducibility, genetic heterogeneity, drug sensitivity, drug resistance and core applications.

## 2 How to achieve and cultivate the PCTS

The preparation and incubation of PCTS closely resemble the methods used for slices derived from the corresponding healthy organs. The selection of the optimal methodology must be determined by the characteristics of the tissue, including size, stiffness, and cellular or ECM composition.

### 2.1 Pretreatment and section

Optimal PCTS preparation involves three sequential phases: transportation, pretreatment and sectioning. Freshly resected tumors should be immediately immersed in ice-cold organ preservation solution (e.g., Belzer UW) or cell culture medium supplemented with standard energy substrates to maintain tissue viability. Hanks’ balanced salt solution may be used for less than 24 hours of storage (Nagaraj et al., 2018). Prior to sectioning, tumor specimens are pretreated according to size, shape, and consistency (de Graaf et al., 2010), with common steps including pre-cutting and embedding. For small or soft samples, such as brain tissue, embedding in agarose (2%, w/v) (Rosales Gerpe et al., 2018) or hydrogels (7–7.5 wt%) (Blomberg et al., 2024) is typically adopted. Furthermore, pre-cutting is the standard recommendation when the tissue diameter exceeds approximately 3 mm.

Sectioning is the final and most critical step. At present, Krumdieck and Brendel–Vitron slicers remain the most widely utilized instruments. The slicer that was originally developed by Krumdieck et al. in 1980 for liver slice preparation introduced a revolutionary advancement. The instrument allows the adjustment of the cutting frequency and advancement speed according to tissue compliance, enabling the generation of adequately thin and accurate slices across diverse sample types (Krumdieck, 2013). The Brendel–Vitron slicer is a semiautomated instrument that was developed based on the Krumdieck instrument in 1998; it incorporates a rotating blade and simultaneously oxygenates/circulates slicing buffer via integrated gas delivery to maintain tissue viability (Price et al., 1998). Although newer slicers such as the McIlwain tissue chopper (Adler-Levy et al., 2019) and Leica VT1200 S (Jiang et al., 2017; Misra et al., 2019; Hughes et al., 2024; Collins et al., 2025) have been developed, the Krumdieck slicer is still widely used for preparing PCTS from breast cancer (Carranza-Torres et al., 2015), prostatic adenocarcinomas (Zhao et al., 2010), and various tumor xenografts (Roife et al., 2016) because of its established reliability (**Figure 1**).

**Figure 1:**
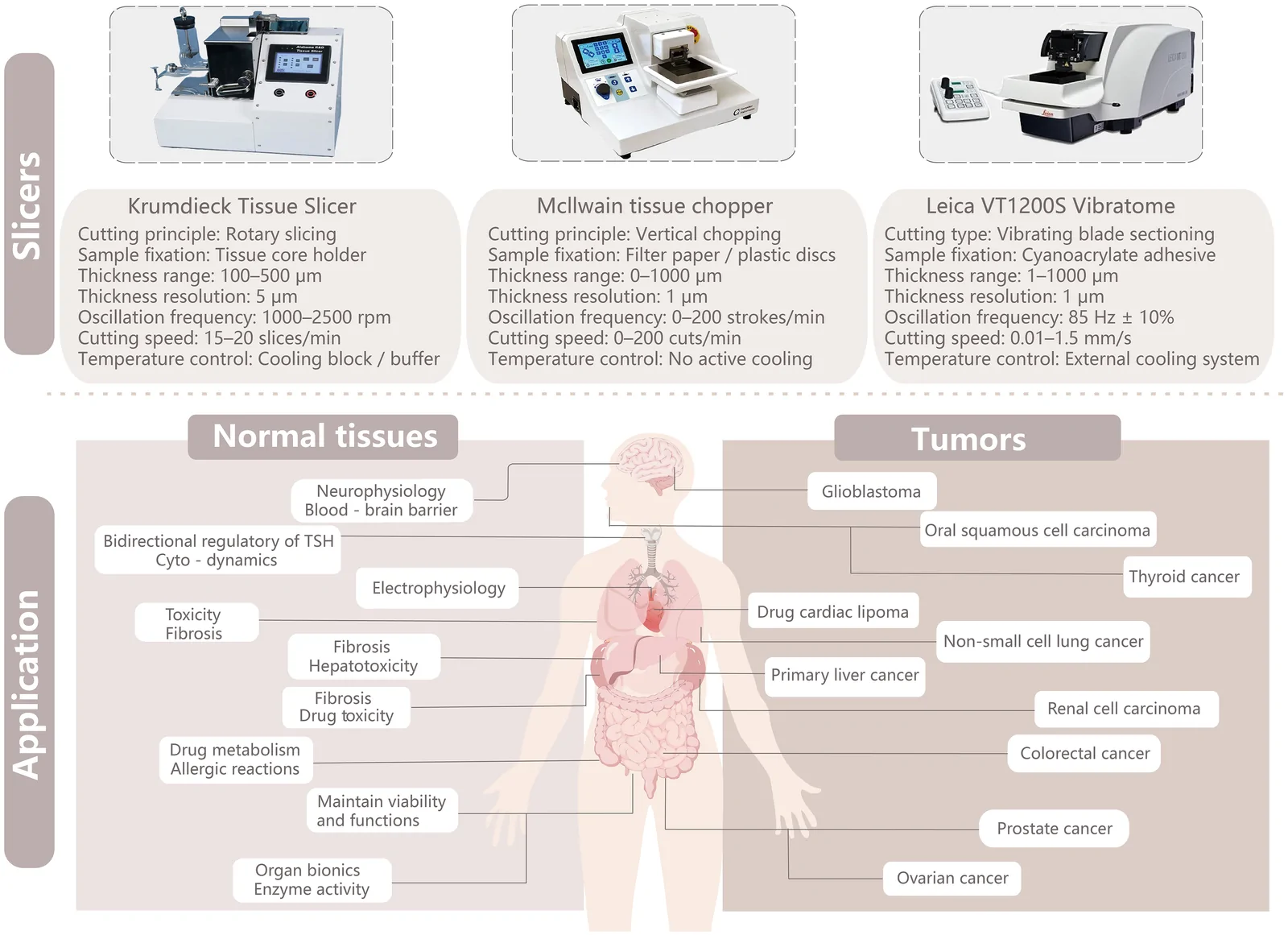
Three widely used slicers and their applications in preparing normal and tumor tissue slices from different types of organs. The Krumdieck slicer was developed in the 1980s and is still widely used. Currently, semi-automated instruments like the McIlwain tissue chopper and Leica VT1200 S are becoming more prevalent. These slicers are used to prepare normal organotypic tissue slices for assessing drug toxicity in antitumor therapy, and PCTS for efficacy discovery across a relatively comprehensive spectrum of tumor types.

The selection of the slicer type and slicing parameters, including the fixation method, sectioning speed, oscillation amplitude, and step size, should be tailored to the tumor type and size to minimize the loss of PCTS activity and function. This step is critical for experimental reproducibility. Krumdieck and Brendel–Vitron slicers are poorly suited for small or irregularly shaped tissues, as they require preprepared cylindrical specimens, which substantially increases tissue loss (Zimmermann et al., 2009). For small samples, adhesive-assisted fixation during sectioning is often necessary, and the Leica VT1200 offers clear advantages in this regard (Zimmermann et al., 2009; Smirnova et al., 2025). Moreover, the parameters of the sections, especially the sectioning speed, oscillation amplitude and step size, are also affected by the stiffness and density of different types of tumors. For instance, Young’s modulus of hepatocellular carcinoma has been reported to range from 4.5 to 265 kPa (Massey et al., 2024). Greater degrees of liver fibrosis result in denser and more brittle tissue, which is prone to fragmentation during sectioning. Therefore, 300 μm is widely accepted as a favorable slice thickness for this tumor type, but the exact thickness should be tailored to the severity of underlying cirrhosis (Zhang et al., 2022). In contrast, Young’s modulus of glioblastoma ranges from 70 Pa to 13.5 kPa, and this tissue features an overall soft texture that is susceptible to deformation during cutting. Accordingly, the minimum thickness of a glioblastoma PCTS can be as low as 250 μm (Horowitz et al., 2020). However, due to the reasonable slicing parameters of existing slicers, they are applicable to various types of tumor tissues.

### 2.2 Incubation

Successful long-term culture of PCTS is essential for investigating the evolution of tumor heterogeneity and tracking disease progression. Oxygen and nutrients are essential for cell proliferation and metabolism. PCTS from pancreatic, breast, prostate, and lung cancers exhibit distinct cellular responses when they are cultured under normoxic (21% O_2_), hyperoxic (90% O_2_), or hypoxic/anoxic conditions (<5% O_2_) (van Geer et al., 2009; Rebours et al., 2013). However, a study in which ex vivo pancreatic tumor slices were cultured revealed no quantitative differences in tissue viability, proliferative activity, or pS6 expression between paired samples maintained under normoxic and hyperoxic conditions (Misra et al., 2019). These findings suggest that oxygen levels in PCTS cultures should be tailored to the intrinsic oxygen consumption characteristics of each organ rather than applied uniformly. Given the critical role of the oxygen concentration in PCTS, mathematical models have been developed to predict physiologically relevant oxygen gradients within these systems (Chidlow et al., 2022).

Nutrients derived from complete culture medium, including L-glutamine, fetal bovine serum (FBS) and buffering agents, is crucial for activity. In addition, other supplements must be tailored to the specific tumor type and experimental objectives. For example, insulin–transferrin–selenium is used to preserve the metabolic functions of human liver tissue and associated tumors (Wu et al., 2018), and immune and inflammatory factors help reconstruct the tumor immune microenvironment (Jagatia et al., 2023).

Oxygen tension, nutrient supply, and metabolic waste removal are key determinants of PCTS viability. Thus, a culture platform capable of supporting both gas and nutrient exchange is essential. The widely used platform is a multiwell cell culture plate (Parajuli and Doppler, 2009; Zhang et al., 2022; Collins et al., 2025), which provides sufficient metabolite exchange and operational simplicity but fails to replicate the physiological shear stress induced by body fluids. Platforms incorporating orbital shakers or rocking incubators introduce dynamic flow to mimic circulation *in vivo* and increase the limited viability of PCTS (Hughes et al., 2024). However, Zhang et al. demonstrated that static filter cultures maintained higher cellular viability than floating or rotating cultures (Zhang et al., 2022). Recent advances have increasingly utilized perfusion and rotating bioreactor systems for PCTS culture. Among the dynamic platforms, microfluidic chips excel because of their precise control over mechanical forces, structural configurations, and chemical gradient delivery at cellular resolution (Ingber, 2022). Typical microfluidic chips for PCTS incubation are made of polydimethylsiloxane and glass substrates (van Midwoud et al., 2012). These chips enable slices to remain viable for up to two weeks (Mauleon et al., 2012). Beyond sustaining viability (Vega et al., 2023; Delong et al., 2024), microfluidic architectures support diverse applications, such as the coculture of multi-organic PCTS (de Hoyos-Vega et al., 2020; Parsian et al., 2022), high-throughput drug response profiling (Rodriguez et al., 2020), living imaging (Rafiei et al., 2022), and spatially resolved electrical/biochemical stimulation (Dorrigiv et al., 2021; Shaner et al., 2023; Hu et al., 2024).

## 3 Applications in basic research

From the 1980s to the present, the hallmarks of cancer, proposed by Robert A. Weinberg and Douglas Hanahan, have guided fundamental investigations and clinical procedures (Hanahan, 2022). Organotypic tissue slice models have attracted significant attention for investigating the roles of gene damage and mutation in tumorigenesis and proliferation, invasion and metastasis, tumor-promoting inflammation and immune evasion (**Figure 2**).

**Figure 2:**
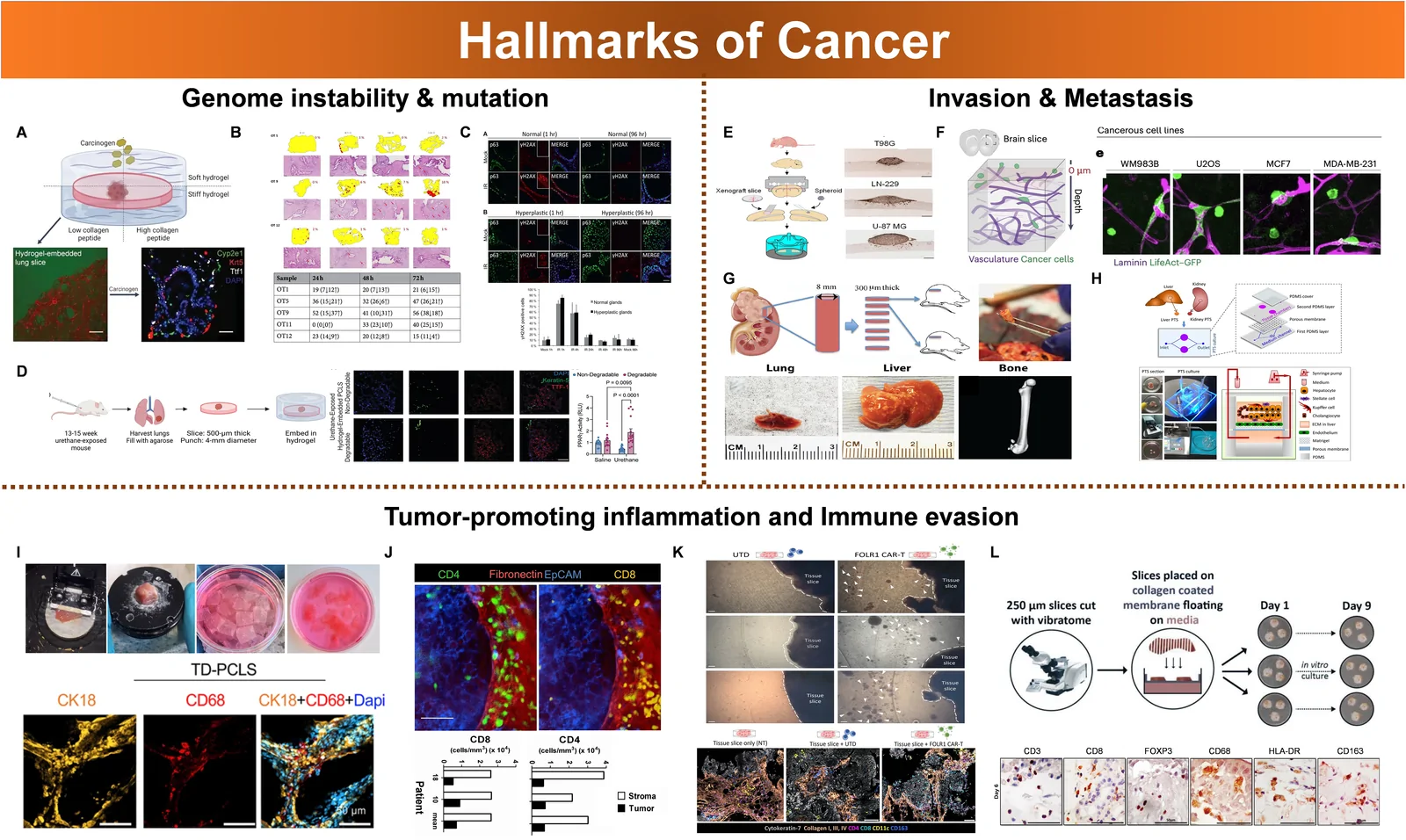
Leveraging PTS to study the hallmarks of cancer in basic research. **(A–D)** PTS from healthy donors and tumor patients were cultured *in vivo* (mouse models) or *in vitro* and then analyzed using comet assay and multi omics to evaluate DNA damage and mutation. **(E–H)** PCTS are applied to research invasion and metastasis. **(I–L)** Researchers can directly visualize the infiltration and activity of immune cells such as cytotoxic T cells, antigen presenting cells, macrophages, dendritic cells, and CAR T cells within the native tumor microenvironment by using PCTS. **A**. Blomberg et al., 2024, reproduced from Mendeley Data (https://data.mendeley.com/datasets/gm93j5r9fw/3), licensed under CC BY 4.0 International (https://creativecommons.org/licenses/by/4.0/). **B**. Ghaderi M et al., 2020, reproduced from Ghaderi M et al. Genome-wide transcriptome profiling of ex-vivo precision-cut slices from human pancreatic ductal adenocarcinoma. Scientific Reports, 2020, 10: 9070, https://doi.org/10.1038/s41598-020-65911-3, licensed under CC BY 4.0 International (https://creativecommons.org/licenses/by/4.0/). **C**. Af Hällström et al., 2014, reproduced with permission from Elsevier, 2014. Original source: af Hällström TM et al. A tissue graft model of DNA damage response in the normal and malignant human prostate. J Urol. 2014;191:842-849, https://doi:10.1016/j.juro.2013.09.007. **D**. Hauer C et al., 2025, reproduced from Hauer C et al. Hydrogel-embedded precision-cut lung slices support ex vivo culture of in vivo-induced premalignant lung lesions. Physiol Rep. 2025;13:e70459, https://doi.org/10.14814/phy2.70459, licensed under CC BY 4.0 International (https://creativecommons.org/licenses/by/4.0/). **E**. Sidorcenco V et al., 2020, reproduced from Sidorcenco V et al. Glioblastoma Tissue Slice Tandem-Cultures for Quantitative Evaluation of Inhibitory Effects on Invasion and Growth. Cancers. 2020;12:2707, https://doi.org/10.3390/cancers12092707, licensed under CC BY 4.0 International (https://creativecommons.org/licenses/by/4.0/). **F**. Uroz M et al., 2024, reproduced with permission from Springer Nature. Uroz M et al. Differential stiffness between brain vasculature and parenchyma promotes metastatic infiltration through vessel co-option. Nat Cell Biol. 2024;26:2144-2153, https://doi.org/10.1038/s41556-024-01532-6. **G**. Thong AE et al., 2014, reproduced with permission from Elsevier. Thong AE et al. Tissue slice grafts of human renal cell carcinoma: an authentic preclinical model with high engraftment rate and metastatic potential. Urol Oncol. 2014;32(1):43.e23-30, https://doi.org/10.1016/j.urolonc.2013.05.008 **H**. Tian H et al., 2020, reproduced from Tian H et al. A Novel Tissue-Based Liver-Kidney-on-a-Chip Can Mimic Liver Tropism of Extracellular Vesicles Derived from Breast Cancer Cells. Biotechnol J. 2020;15:e1900107, https://doi.org/10.1002/biot.201900107, licensed under CC BY 4.0 International (https://creativecommons.org/licenses/by/4.0/) **I**. Mansouri S et al., 2025, reproduced from Mansouri S et al. Living human lung slices for ex vivo modelling of lung cancer. JCI Insight. 2025;10:e190703, https://doi.org/10.1172/jci.insight.190703, licensed under CC BY 4.0 International (https://creativecommons.org/licenses/by/4.0/). **J**. Salmon H et al., 2012, reproduced from Salmon H et al. Matrix architecture defines the preferential localization and migration of T cells into the stroma of human lung tumors. J Clin Invest. 2012;122:899-910, https://doi.org/10.1172/jci45817, licensed under CC BY 4.0 International (https://creativecommons.org/licenses/by/4.0/) **K**. Durante V et al., 2025, reproduced from Durante V et al. Precision-cut tumor tissue slices, a novel tool to study the tumor microenvironment interactions with chimeric antigen receptor (CAR) T cells. PLoS One. 2025;20:e0327322, https://doi.org/10.1371/journal.pone.0327322, licensed under CC BY 4.0 International (https://creativecommons.org/licenses/by/4.0/). **L**. Jiang X et al., 2017, reproduced from Jiang X et al. Long-lived pancreatic ductal adenocarcinoma slice cultures enable precise study of the immune microenvironment. Oncoimmunology. 2017;6:e1333210, https://doi.org/10.1080/2162402x.2017.1333210, licensed under CC BY 4.0 International (https://creativecommons.org/licenses/by/4.0/).

### 3.1 Roles of gene damage and mutation in tumorigenesis and proliferation

Tumorigenesis and proliferation are driven by genomic mutations and DNA damage (Hobor et al., 2024). In addition to their high architectural complexity and genetic heterogeneity, tissue slices have a distinct advantage over conventional cell-based models because they allow the concurrent acquisition of normal and matched tumor tissues, facilitating direct side-by-side comparisons. For example, PCTS from healthy donors and prostate cancer patients were grafted either subcutaneously or beneath the renal capsule in mice. After cultivation, renal capsule grafts exhibited nearly 100% proliferation rates and full vascularization, indicating the utility of this site for studying DNA damage in tumors (Af Hällström et al., 2014). The comet assay remains a standard method for assessing DNA damage at the cellular level. Recently, 100-μm-thick breast cancer explants were used as an alternative tool for evaluating DNA damage and the effects of chemicals on intact tissue (García-Vielma et al., 2025). Multiomics analysis reveals comprehensive molecular profiles (e.g., transcriptomic, proteomic, and metabolomic) of tumors (Baysoy et al., 2023). Multiomics analysis based on the PCTS also revealed that the expression of a small number of genes was significantly changed from baseline (Ghaderi et al., 2020). Critically, hydrogel-embedded PCTS exposed to vinyl carbamate successfully modeled lung cancer premalignancy, establishing a foundation for ex vivo studies of carcinogenesis and chemoprevention (Blomberg et al., 2024; Hauer et al., 2025).

### 3.2 Invasion and metastasis

Tumor invasion and metastasis are spatiotemporally dynamic processes that involve intricate cell–cell and cell–ECM interactions within the TME. Conventional ex vivo models lack physiological relevance for metastasis, as they cannot recapitulate the complex *in vivo* interplay among primary tumors, the circulatory system, and distal organs (Blazquez and Pukrop, 2017; Spennati et al., 2021; Blazquez et al., 2024). As a method to address these limitations, PCTS have been co-cultured with tumor cells to visualize how tumor cells invade proximal tissue interfaces (Eisemann et al., 2018; Sidorcenco et al., 2020; Ravin et al., 2023). Human PCTS can be implanted into murine models and more faithfully recapitulate metastasis, enabling detection by histology and advanced imaging (Thong et al., 2014; Valta et al., 2014). Murine tissue slices loaded with pro-metastatic signals have also been used to study invasion and metastasis in coculture with human PCTS (Koch et al., 2014; Decotret et al., 2023). In a recent study, Liu et al. employed a microfluidic platform to culture rat tissue slices derived from the lung, liver, and kidney and showed that breast cancer-derived extracellular vesicles exhibit distinct organotropism (Tian et al., 2020).

### 3.3 Tumor-promoting inflammation and immune evasion

Significant advancements in immunotherapy for malignant tumors have been achieved through in-depth explorations of tumor-mediated immunomodulatory mechanisms within the TME (Greten and Grivennikov, 2019; Wu et al., 2026d). Compared with cellular and animal models, PCTS more accurately replicate physiological immune cell–tumor cell interactions in the TME. This fidelity enables essential investigations of immunosuppressive mechanisms to accelerate clinical translation (Peranzoni et al., 2017; Wu et al., 2025). CD8^+^ cytotoxic T cells are the main effectors of anticancer immunity (Raskov et al., 2021). Following continuous culture, persistent populations of CD3^+^/CD8^+^/FOXP3^+^ T cells and CD68^+^/CD163^+^/HLA-DR^+^ macrophages were observed in PCTS (Jiang et al., 2017). Moreover, key features of the tumor-associated ECM are retained, allowing the assessment of immune-related protein stability as well as T-cell infiltration and activity (Salmon et al., 2012; Du et al., 2023). Tumor-associated monocytes exhibit dual functions and contribute to both immunosuppression and proinflammatory processes. Studies using PCTS of renal cell carcinoma revealed that PD-L1^+^/HLA-DR^+^ cells exhibit a macrophage-like or dendritic cell-like morphology and promote tumor inflammation and immune tolerance (Kusmartsev et al., 2022). Recently, Herbel et al. demonstrated the potential of PCTS as an effective ex vivo model for investigating CAR-T-cell infiltration and interactions within the TME (Durante et al., 2025). However, the reliability of the current experimental results still requires improvement, mainly because the immune and inflammatory cells are largely restricted to those present at the time of PCTS preparation, with no sustained supply from circulating immune cells.

## 4 A novel preclinical model for investigating cancer therapy

In addition to surgical resection, cancer treatments can be categorized as pharmacological or nonpharmacological. Pharmacological approaches include chemotherapy or cytotoxic drugs, small-molecule targeted inhibitors (SMIs), and immune checkpoint inhibitors (ICIs). Nonpharmacological interventions include radiotherapy, gene therapy, oncolytic virotherapy, and therapeutic vaccines. Before advancing to clinical trials, the efficacy of novel therapeutics must be evaluated in animal models (Teicher, 2006). However, the FDA no longer requires animal testing of a potential drug before clinical trials. PCTS represent a potential strategy to bridge this translational gap.

### 4.1 Applications in drug screening

Chemotherapy agents are typically used in different stages of tumor progression because they reduce the survival, proliferation and metabolism of tumor cells (Luo et al., 2017). PCTS were employed in 1995 to study the metabolism of 7-ethoxycoumarin in the rat liver and lung (Price et al., 1995) and were subsequently applied to evaluate drug sensitivity, metabolism and toxicity in diverse human cancers. These different types of tumors were derived from different systems, such as the nervous system (Merz et al., 2013), head and neck (Bohlen et al., 2019; Riley et al., 2019), respiratory system (Lauenstein et al., 2014; van Rijt et al., 2015; Watson et al., 2016), digestive system (Koerfer et al., 2016; Karkampouna et al., 2018; Sönnichsen et al., 2018; Hughes et al., 2024; Cros et al., 2025; Etzold et al., 2025; Mönch et al., 2025), reproductive and urinary systems (Chakrabarty et al., 2022; Dong et al., 2023), and individual tumors, such as breast cancer (Chakrabarty et al., 2022; Ladan et al., 2022; Carranza-Rosales et al., 2023; Komar et al., 2025). Collectively, these studies indicate that PCTS exhibit reproducible phenotypic changes that are highly concordant with *in vivo* and clinical drug responses, suggesting their utility as a predictive ex vivo platform for assessing the therapeutic response and precision oncology applications. By preserving tumor heterogeneity, metabolic characteristics, and complex microenvironmental interactions, PCTS provide a physiologically relevant platform for investigating therapeutic efficacy, resistance mechanisms, and novel therapeutic targets, which are increasingly recognized as critical determinants of tumor progression and the treatment response (Wu et al., 2026a).

Compared with conventional chemotherapies, SMIs and ICIs provide enhanced targeting and reduced systemic toxicity. For SMIs that are already in clinical use or advanced clinical trials, such as cetuximab, sorafenib (Donnadieu et al., 2016), dactolisib (Hörnschemeyer et al., 2022), darapladib (Wang et al., 2020), regorafenib (Zhang et al., 2022; Pham et al., 2024), gefitinib (Parker et al., 2013; Parker et al., 2018), olaparib (Zhang et al., 2019; Elsesy et al., 2023; Guffanti et al., 2024; Pham et al., 2024), cabozantinib (Zhao et al., 2017), emtansine (Liu et al., 2025), staurosporine (Lim et al., 2018), palbociclib (Vilgelm et al., 2019) and sunitinib (Roelants et al., 2020), PCTS enable precise assessments of drug efficacy and side effect profiles across diverse patient populations (Estes et al., 2007; Niessner et al., 2023). Furthermore, since the reliability of the use of the PCTS in evaluating SMI efficacy is comparable to that in murine models, they are being increasingly used as an essential tool for assessing the efficacy of treatment during the development of novel SMIs (**Table 2**).

**Table 2:** Tumor precision-cut slices culture system for pharmacological therapy screening.

Anatomical classification	Tumor type	Chemical agents	SMIs*	ICIs**
Nervous system	Glioblastoma	Temozolomide (Merz et al., 2013)	Darapladib (Wang et al., 2020) Dactolisib (Hörnschemeyer et al., 2022) Gefitinib (Parker et al., 2013; Parker et al., 2018)	--
Head and neck	Squamous cell carcinoma	Fluorouracil (Bohlen et al., 2019)	Cetuximab and Sorafenib (Donnadieu et al., 2016)	--
	Thyroid cancer	Etoposide (Riley et al., 2019)	SP600125 (Riley et al., 2019)	--
Respiratory system	Lung cancer	Cisplatin (Lauenstein et al., 2014; van Rijt et al., 2015)	--	Pembrolizumab (Fan et al., 2021) Nivolumab (Junk et al., 2021; Bargmann et al., 2025)
Digestive system	Esophageal squamous cell carcinoma	Fluorouracil (Koerfer et al., 2016)	--	--
	Gastric cancer	Fluorouracil (Etzold et al., 2025) Cisplatin (Koerfer et al., 2016)	--	Nivolumab (Zhang et al., 2022; Hennig et al., 2025)
	Hepatocellular carcinoma	Doxorubicin (Karkampouna et al., 2018)	Regorafenib (Zhang et al., 2022)	Nivolumab (Collins et al., 2025) PD-L1 and CTLA-4 antibody (Sivakumar et al., 2019)
	Cholangiocarcinoma	Emtansine (Liu et al., 2025)	--	--
	Colon cancer	Fluorouracil (Sönnichsen et al., 2018)	Dabrafenib (Sivakumar et al., 2019)	Pembrolizumab (Martin et al., 2019)
	Pancreatic cancer	Gemcitabine (Hughes et al., 2024) Everolimus (Cros et al., 2025)	Olaparib and Regorafenib (Pham et al., 2024) Staurosporine (Lim et al., 2018) XP-524 (Principe et al., 2022)	--
Reproductive system	Ovarian cancer	Cisplatin (Dong et al., 2023)	Olaparib (Guffanti et al., 2024)	Durvalumab (Mirza et al., 2024)
Urinary system	Renal cell carcinoma	--	Cabozantinib (Zhao et al., 2017) Sunitinib (Roelants et al., 2020)	Nivolumab (Stenzel et al., 2021)
	Prostate cancer	Cisplatin (Chakrabarty et al., 2022)	Olaparib (Zhang et al., 2019; Elsesy et al., 2023)	Nivolumab (Chang et al., 2025)
Others	Breast cancer	Paclitaxel (Komar et al., 2025) Fluorouracil (Carranza-Rosales et al., 2023) Cisplatin (Chakrabarty et al., 2022)	SB590885 (Sivakumar et al., 2019)	Pembrolizumab (Sivakumar et al., 2019)
	Melanoma	--	Palbociclib (Vilgelm et al., 2019) Encorafenib and Binimetinib (Niessner et al., 2023)	Nivolumab (Voabil et al., 2021)

^*^SMIs, small–molecule inhibitors. ^**^ICIs, immune checkpoint inhibitors. This table compiles high-quality literature on the application of Tumor precision-cut slices in drug screening at home and abroad. The table mainly focuses on the screening of three types of drugs: chemotherapy drugs, small molecule inhibitors, and immune checkpoint inhibitors, and conducts research from various systems of the human body.

TME heterogeneity remains a major limitation to the efficacy of immunotherapy in various solid tumors. PCTS not only maintain the complex cellular and ECM components of an individual patient’s tumor but also enable the assessment of drug efficacy and the emergence of resistance throughout long-term culture. Researchers have already widely achieved responses to single-agent ICIs, such as pembrolizumab and nivolumab, using PCTS from multiple cancer types, including lung cancer (Junk et al., 2021), colorectal cancer with hepatic metastasis (Martin et al., 2019), renal cell carcinoma (Stenzel et al., 2021), prostate cancer (Chang et al., 2025), ovarian cancer (Mirza et al., 2024) and melanoma (Voabil et al., 2021). Given the limitations of monotherapy, combination regimens incorporating ICIs with other agents are being increasingly explored. PCTS provide a powerful platform for screening optimal drug combinations by quantifying key indicators, such as T-cell activation, shifts in immune cell metabolism, and the degree of on-target tissue damage (Sivakumar et al., 2019; Fan et al., 2021; Principe et al., 2022; Bargmann et al., 2025).

### 4.2 Development of nonpharmacological therapeutic methods using PCTS

The development of radiotherapy involves hypersensitivity, radiation resistance, dose optimization, the biological response and protection against radiation injury (Verginadis et al., 2025). In lung cancer, the ability of LGM2605 to protect against radiation damage was evaluated in normal lung tissue slices exposed to high-dose proton beams (Velalopoulou et al., 2017). Theresa Suckert et al. successfully utilized brain and HNSCC-derived slices to assess the toxicity of proton irradiation (Suckert et al., 2020). Current advancements focus on combining radiotherapy with ICIs and SMIs in individualized treatment (Rodriguez-Berriguete et al., 2023). HNSCC-derived PCTS enable a discrimination between patients who are sensitive to ionizing radiation and those who are resistant to it and shows promise for predicting the response to cisplatin (Capala et al., 2023).

Biological therapies, including adoptive cellular therapies and oncolytic viruses or cancer vaccines, constitute another field in oncological treatment (Papież and Krzyściak, 2021). PCTS of breast cancer were first shown to be useful for evaluating adenovirus and oncolytic viral efficacy in 2005 (Stoff-Khalili et al., 2005). Within five years, PCTS models quickly became powerful models for determining the specificity and potency of biological therapies across multiple malignancies (Relph et al., 2020; LaRocca et al., 2021; Runge et al., 2022; van de Merbel et al., 2022). Adoptive cellular therapy employs engineered immune cells expressing antigen-specific T-cell receptors or chimeric antigen receptors to increase tumor recognition and elimination (Klebanoff et al., 2023). However, no published studies have described their application for evaluating cellular therapies in solid tumors.

The use of PCTS for evaluating nonpharmacological therapies is still underexplored. With respect to radiotherapy, the disconnect between patient-relevant therapeutic doses and PCTS-tolerable doses severely limits the translational relevance of preclinical data. In the context of cellular therapy, two additional challenges arise. First, the action of cellular therapies in solid tumors is still poorly defined (Vo et al., 2025), making the establishment of appropriate controls for PCTS-based assays challenging. The other is that PCTS, while uniquely capable of preserving tumor heterogeneity—a known hurdle to cell therapy efficacy—suffer from inherently low reproducibility as a trade-off.

## 5 Discussion and perspectives of the clinical translation of PCTS

Due to their widespread use in fundamental and preclinical research, PCTS have also been explored to support clinical diagnosis and treatment (Gao et al., 2025; Hennig et al., 2025). We believe that PCTS will ultimately impact precision clinical diagnosis, therapeutic monitoring, and drug screening. For tumor diagnosis and monitoring, we propose that PCTS should move beyond simple biochemical readouts, evolve toward spatially resolved multi-omics approaches anchored in tissue viability, and the development of high-resolution real-time imaging techniques compatible with thick-section histology should be prioritized. While PCTS effectively overcome the key drawbacks of conventional models, namely, TME heterogeneity, immune variability, and temporal drug response profiles, substantial challenges remain in translating this platform into routine clinical practice. These issues include maintaining tissue viability and inter-tumor reproducibility over time, as well as establishing standardized operational workflows.

### 5.1 Effective long-term cultivation and cryopreservation

Sustained PCTS viability and protumorigenic function hinge on two parallel priorities that are both indispensable for downstream readouts: tailoring the oxygen concentration, nutrients, and medium to each tumor type and actively maintaining the protumorigenic microenvironment. However, the TME, which is characterized by acidosis, hypoxia, and metabolic imbalance, actively fuels malignancy (Sureka et al., 2026). Paradoxically, these protumorigenic conditions, which are advantageous *in vivo*, become detrimental to PCTS maintenance ex vivo. Consequently, achieving a balance between sustaining viability and preserving tumor-relevant function remains a formidable challenge.

Standardized cryopreservation and thawing protocols for PCTS remain underdeveloped, limiting their clinical popularization. The fundamental principle for cryopreserving and thawing viable cells involves slow freezing followed by rapid rewarming; this principle generally applies to PCTS as well (Kasper et al., 2011). However, the composition of cryopreservation solutions is different. Tissue vitrification solutions are typically employed to minimize ice crystal formation and protect cellular integrity within PCTS (de Graaf et al., 2002; Zhang et al., 2020; Zhang et al., 2022). Alternatively, solutions similar to those used for cell cryopreservation, such as culture medium supplemented with DMSO, can be used (Kasper et al., 2011). Precise control of temperature and timing during the cryopreservation process is critical. Researchers developed a computer-controlled system for freezing nontumor tissues to achieve this goal and maintained post thaw viability between 60% and 100% (Maas et al., 2000). The Bischof laboratory explored an “ice-free” cryopreservation approach. In this method, rat liver PCTS were successfully cultured for 3 days after vitrification and rewarming, and sufficient functionality was retained for drug toxicity testing (Ramesh et al., 2026). Although this method was tested only with rat tissues, it also allowed the possibility of developing a similar protocol for human PCTS for oncologists.

### 5.2 Standardized operation based on personalized parameters for patients

Different tissue origins and preparation procedures have been used to create a platform with intrinsic advantages and limitations in the field of anticancer drug discovery or clinical translation, and thus standardized workflows and dependable readouts are critical. **Table 3** summarizes the preparation parameters currently established for PCTS derived from distinct tumor types. Future studies should prioritize the expansion of clinical inclusion criteria and the comprehensive assessment of drug metabolism and biotoxicity, which will be key to advancing the clinical applicability of PCTS.

**Table 3:** Summary of preparation and evaluation parameters for PCTS across different solid tumors.

Cancer type	Thickness	Cultivation time	Slicing speed	Frequency	Evaluation criteria	Ref.
Glioma	300–500 μm	Acute (hours)	--	--	Histological evaluation Immunofluorescence staining PpIX fluorescence assay	(Kirby et al., 2021)
Head and neck squamous cell carcinoma	300 μm	5 d	0.6 mm/s	2 mm	Histological evaluation TUNEL assay EdU incorporation assay	(Capala et al., 2023)
Lung cancer	300 μm	30 d	8 µm/s	–	Live–dead viability staining	(Rosales Gerpe et al., 2018)
Liver cancer	250 μm	6 d	0.5–1.5 mm/s	2–3 mm	Cell viability/proliferation assay	(Kenerson et al., 2021)
Colorectal cancer	250 μm	7 d	0.5–1.5 mm/s	2–3 mm	MTS assay Histological evaluation	(Kenerson et al., 2020)
Breast cancer	300 μm	14 d	0.6 mm/s	3 mm	Histological evaluation TUNEL assay	(Naipal et al., 2016)
Pancreatic cancer	300 μm	6 d	0.04 mm/s	1 mm	Resazurin viability assay Histological evaluation	(Braun et al., 2021)
Bladder cancer	300 μm	2 d	0.4 mm/s	3 mm	Tumor TACS *in situ* apoptosis detection	(Relph et al., 2020)

Color scale ranges from green (maximum positive value) to red (maximum negative value). The texture of tumor tissues in different organs varies greatly, so there is no uniform and fixed optimal slice thickness for PCTS preparation. Thick slices in the central area are prone to ischemic damage, while thin slices can damage cell structures and cause tissue fragmentation. This table sorts out the PCTS modeling parameters of common solid tumors, covering slice thickness, maximum *in vitro* culture duration and corresponding tissue quality evaluation methods. The evaluation dimensions include histological integrity, apoptosis level and proliferation activity, providing literature support for the standardized construction of PCTS models of different cancer types.
